# Effects of bradykinin on the survival of multiterritory perforator flaps in rats

**DOI:** 10.1186/s12957-019-1570-3

**Published:** 2019-02-27

**Authors:** Jieke Wang, Encheng Ji, Chen Lin, Long Wang, Li Dai, Weiyang Gao

**Affiliations:** Department of Hand and Plastic Surgery, The Second Affiliated Hospital and Yuying Children’s Hospital of Wenzhou Medical University, The Second School of Medicine, The Second Clinical Medical College of Wenzhou Medical University, Wenzhou Medical University, No. 109, Xue Yuan Road (West), Lucheng District, Wenzhou, 325000 China

**Keywords:** Bradykinin, Perforator flap, Apoptosis, Autophagy, Angiogenesis

## Abstract

**Background:**

Bradykinin, a vasoactive peptide, has many biological functions. For example, it accelerates angiogenesis. Thus, we studied the effects of bradykinin on the survival of perforator flaps.

**Methods:**

Averagely, 50 male Sprague–Dawley rats were divided into control and bradykinin groups and underwent procedures to the multiterritory perforator flap. Areas of flap survival were tested 7 days later. Flap perfusion was evaluated by laser Doppler imaging. We assessed the extent of autophagy by determining LC3-II/I, Beclin 1, and p62. Flap angiogenesis was assessed by immunohistochemistry and H&E staining. We measured the level of vascular endothelial growth factor (VEGF) protein using western blot. We assessed oxidative stress by measuring the activity of superoxide dismutase (SOD) and malondialdehyde (MDA) levels. The apoptotic index was also evaluated by western blot, and we determined nitric oxide (NO) production using an NO assay kit.

**Results:**

The bradykinin group exhibited significantly larger areas of flap survival, higher blood supply, and more neovascularization. The bradykinin group also had higher SOD activity, higher VEGF expression and NO content, and reduced MDA compared to the control group. Rats treated with bradykinin also had lower levels of apoptosis and autophagy relative to the control group.

**Conclusion:**

Our results suggest that bradykinin promotes the survival of multiterritory perforator flaps by increasing angiogenesis, promoting the release of NO, suppressing apoptosis, reducing oxidative stress, and inhibiting autophagy.

## Background

The skin, a protective barrier, is highly vulnerable to trauma which can cause serious skin defects. A multiterritory perforator flap can be used to cover these skin defects. It is a skin flap that involves a perforator artery of 0.5 mm or greater, and is widely used because of their advantages [1, 2]. For example, when the skin is injured or defected by severe trauma and burn etc., the multiterritory perforator flaps can cover the huge skin defects. However, avoiding necrosis of such flaps remains a challenge. Many studies have proven that necrosis of the perforator flap always occurs at the dynamic and potential territories [3–5]. Previous studies have also demonstrated that inadequate blood supply [6], oxidative stress [7], and cells apoptosis [8] are important factors that lead to flap necrosis. Thus, there is a need to find an effective way to improve the survival of the multiterritory perforator flaps.

Bradykinin, a vasoactive peptide released from precursor kininogens by a protease known as kallikrein [9], is an important component of both acute and chronic inflammatory processes [10, 11]. However, bradykinin performs several other biological functions as well. For example, it increases expression of VEGF via the bradykinin B2 receptor [12]. The levels of VEGF are particularly affected; vascular endothelial cells are stimulated to regenerate and proliferate, thus accelerating angiogenesis [13]. Yoshida et al. [14] recently reported that kallikrein gene delivery weakened apoptosis in ischemia–reperfusion (I/R) injury and myocardial infarction via bradykinin. Bradykinin had anti-apoptotic effects in a model of coronary artery disease [15]. Bradykinin also played an antioxidative role in a rat model of acute hyperglycemia [16], in which it reduced oxidative stress under conditions of hyperglycemia. Moreover, many studies suggest that bradykinin negatively regulates both apoptotic and autophagic responses via the PI3K/Akt signaling pathway [17–19]. Excessive autophagy is detrimental although the process of autophagy is advantageous to cell survival [20, 21]. Inhibition of autophagy could inhibit oxidative stress, suppress apoptosis, and enhance perforator flap survival [8].

Several recent publications have demonstrated that bradykinin stimulates nitric oxide (NO) production [22–24]. NO, the product of nitric oxide synthase (NOS), promotes cell proliferation and angiogenesis [25]. NO also inhibits apoptosis and autophagy by stimulating the PI3K/Akt signaling pathway [19], which is beneficial for flap survival. Therefore, we hypothesized that bradykinin inhibits apoptosis and autophagy by promoting the release of NO, which may also improve flap survival.

The anti-apoptotic functions of bradykinin, with its ability to inhibit autophagy, inhibit oxidative stress, accelerate vascularization, and promote the release of NO, should be beneficial to improve multiterritory perforator flap survival. In this study, we hypothesized that bradykinin enhances the survival of multiterritory perforator flaps and analyzed its role in doing so in rats. We used histological and protein analyses to investigate whether bradykinin had these effects in a multiterritory perforator flap. We hope our study leads to novel strategies to improve flap survival.

## Methods

### Animals

Sprague–Dawley (SD) rats, with a closed group of genetic characteristics [26], were used in this study. Fifty healthy male specific-pathogen-free (SPF) rats weighing 250 to 300 g were purchased from the Wenzhou Medical University (license no. SCXK [ZJ] 2015–0001). All used procedures and animal care conformed to the Health Guidelines National Institutes for the Care and Use of Laboratory Animals. The Wenzhou Medical University Animal Research Committee approved the study (wydw 2014–0015). Rats were housed in separate cages with free access to food and water under standard environmental conditions such as temperature 22–25 °C, humidity 60–70%, and 12-h light:12-h dark cycle. Rats were divided into two groups randomly: the bradykinin group and the saline (control) group. Each group contained 25 rats.

### Flap animal model

Rats were anesthetized with intraperitoneal pentobarbital (60 mg/kg) [8]. Before surgery, we removed dorsal fur with a depilatory cream and electric shaver. A deep circumflex iliac artery (DCIA) flap was made on the right side of each rat dorsum [27]. In the particular flap, there are three vascular territories: the anatomic territory, the dynamic territory, and the potential territory. In this flap model, the anatomic territory has a deep circumflex iliac (DCI), the dynamic territory has an intercostal artery (IC), and the potential territory has a thoracodorsal artery (TD) [8]. The flap position was approximately 2.5 × 11 cm in size, and it was standardized by bony landmarks. Transillumination identified the choke vessel zone (CVZ) between the IC and TD. Next, the flap was sutured into the original position after the TD and IC were ligated.

### Drug administration

Rats in the bradykinin group were injected intraperitoneal bradykinin (150 μg/kg; purity = 98.97%; Medchem Express, Princeton, NJ, USA) [28] 30 min before the procedure. Rats in the control group were injected normal saline at an equal volume for the same days. All rats were housed individually. All rats were sacrificed after 7 days.

### Flap assessment

On postoperative day 7 (POD 7), we took high-resolution photographs of the flap with a digital camera. We measured surviving flap areas by superimposing the photographs onto graph paper. The percentages of the viable area were quantified as follows: (range of survival/total flap size) × 100%.

### Experimental design

Five rats in each group were used for each test method. In details, five rats in each group were used for flap survival observation and Laser Doppler perfusion imaging; five rats in each group were used for hematoxylin and eosin (H&E) and immunohistochemistry (IHC) staining; five rats in each group were used for superoxide dismutase (SOD) activity and malondialdehyde (MDA) content tests; five rats in each group were used for NO content test; and another five rats in each group were used for western blot.

### Hematoxylin and eosin staining

A sample (1 × 1 cm) from each flap CVZ [27] was collected after the sacrifice of rats on POD 7, and routine procedures of H&E staining kit (Solarbio Science & Technology, Beijing, China) were performed. The thickness of the flap tissue was measured under a light microscope, and the number of microvessels in each area (/mm^2^) was calculated to understand the condition of microvascular density (MVD).

### Western blot analysis

On POD 7, flaps from the CVZ were stored at − 80 °C. We determined protein concentrations by using the BCA assay (Thermo Fisher Scientific, Rockford, IL, USA). We performed routine procedures. The membranes were incubated with the following primary antibodies: VEGF (1:1000; Abcam, Cambridge, UK), p62 (1:1000; Abcam, Cambridge, UK), Beclin 1 (1:1000; Cell Signaling Technology (CST), Danvers, MA, USA), LC3 (1:1000; Sigma-Aldrich, St. Louis, MO, USA), cleaved caspase-3 (1:1000; CST), Bax (1:1000; CST), BCL-2 (1:1000; CST) and GAPDH (1:2000; Bioworld Technology, St. Louis Park, MN, USA). Next, membranes were incubated with goat anti-rabbit secondary antibody for 2 h. We quantified band intensity using Image Lab (ver. 5.2, Bio-Rad Laboratories, Hercules, CA, USA).

### Immunohistochemistry

On POD 7, samples from the CVZ which were fixed in paraformaldehyde were sectioned into 5 μm slices. Sections were rehydrated in a graded series of ethanol after they were deparaffinized through xylene. Then, sections were immersed in 3% H_2_O_2_ and incubated to saturate nonspecific sites. Last sections were incubated with CD34 (1:50; Abcam, Cambridge, UK) at 4 °C overnight. Sections were imaged at × 100 magnification on an image acquisition system (Olympus, Tokyo, Japan). The number of CD34-positive microvessels was calculated in five dense fields.

### Laser Doppler perfusion imaging

On POD 7, blood perfusion images were obtained by a Laser Doppler instrument (Moor Instruments, Axminster, UK).

### Superoxide dismutase activity and malondialdehyde content

SOD and MDA test kits (Nanjing Jiancheng Biology Institution, Nanjing, China) were used to measure the oxidative stress on the flaps. The flap specimens were obtained from the choke vessel zone on POD 7, weighed, homogenized, and diluted to 10% (volume/volume) on an ice bath. Then, SOD activity and MDA content were detected with the method as reported previously [27].

### NO content assay

NO was assayed spectrophotometrically by measuring the products of NO metabolism using NO content assay kits [27] (Nanjing Jiancheng Biology Institution, Nanjing, China).

### Statistical analyses

We performed statistical analyses using SPSS version 19.0 (SPSS, Chicago, IL, USA). All data are presented as means ± standard errors of the mean (SEMs). We compared data between groups using Student’s independent *t* test and one-way repeated measures analysis of variance. In all analyses, *P* < 0.05 was considered to indicate statistical significance.

## Results

### Surviving area and blood perfusion

**Fig. 1:**
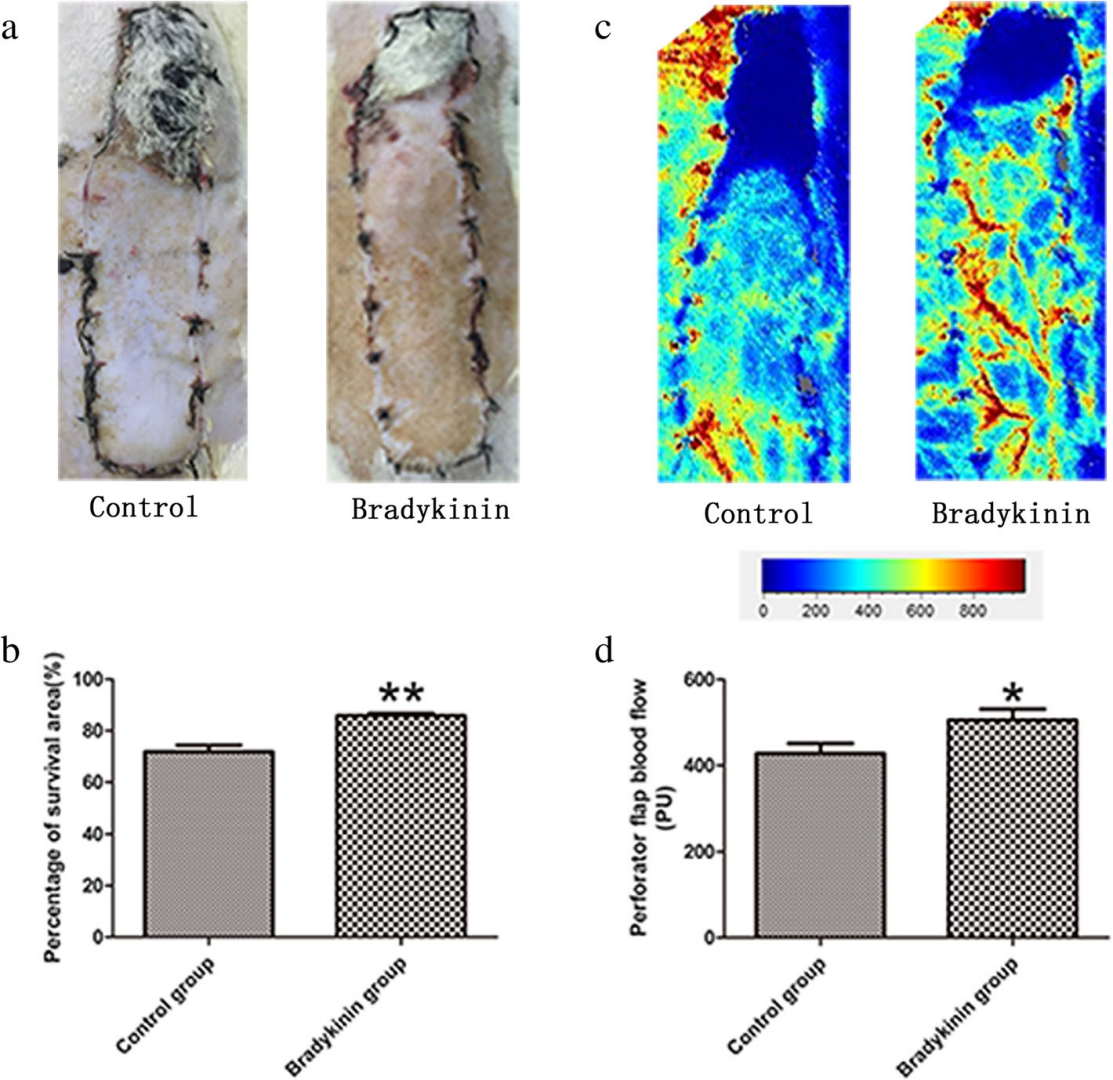
**a** Photographs of the postoperative flaps from the bradykinin and control groups on day 7. **b** The flap survival rate (%) in the bradykinin group (85.83 ± 0.98%) and control group (71.83 ± 2.52%). **c** The perfusion images of a flap on POD 7. Red denotes high perfusion, and blue denotes low perfusion with the scale bar. **d** The perfusion value on POD 7 (control group, 428.38 ± 23.39; bradykinin group, 505.85 ± 25.52). *n* = 5 per group. *P < 0.05, **P < 0.01

The boundary between the surviving and necrotic regions was evident on POD 7 (Fig. 1a). The control group survival rate was 71.83 ± 2.52%, which differed significantly from that of the bradykinin group (85.83 ± 0.98%). Compared to the control group, flap survival was better in the bradykinin group, with less necrosis (*P* < 0.01; Fig. 1b). Laser Doppler images revealed the flap perfusion differences (perfusion units (PU)) were evident on POD 7 (Fig. 1c). Bradykinin improved blood supply at the CVZ compared to the control group (control group, 428.38 ± 23.39; bradykinin group, 505.85 ± 25.52; *P* < 0.05; Fig. 1d).

### Histology

**Fig. 2:**
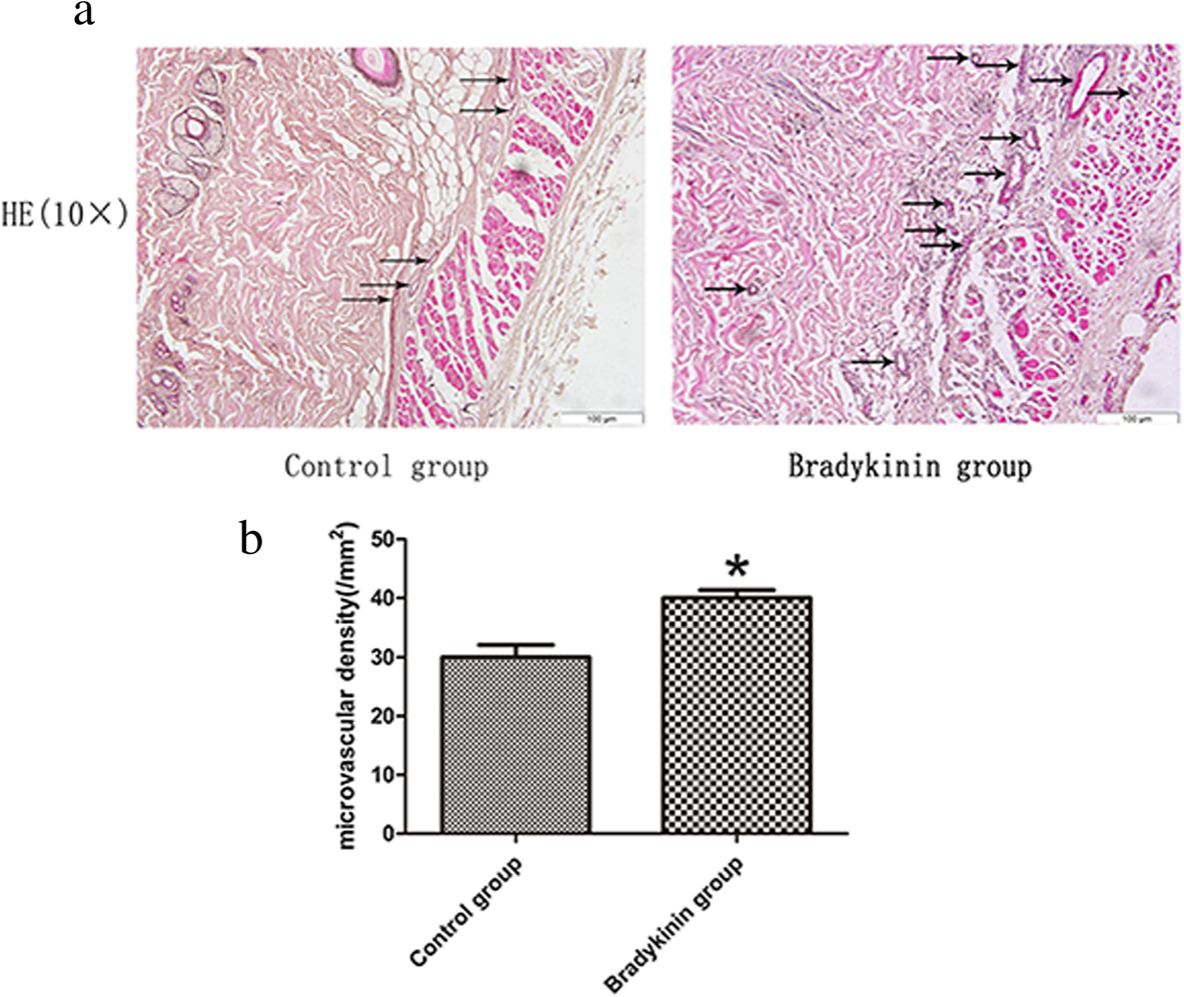
**a** Neovascularization in the bradykinin and control groups (original magnification × 100). **b** The percentage of microvascular density (MVD) in the bradykinin (39.47 ± 1.35/mm^2^) and control (30.38 ± 2.10/mm^2^) groups. *n* = 5 per group. *P < 0.05

**Fig. 3:**
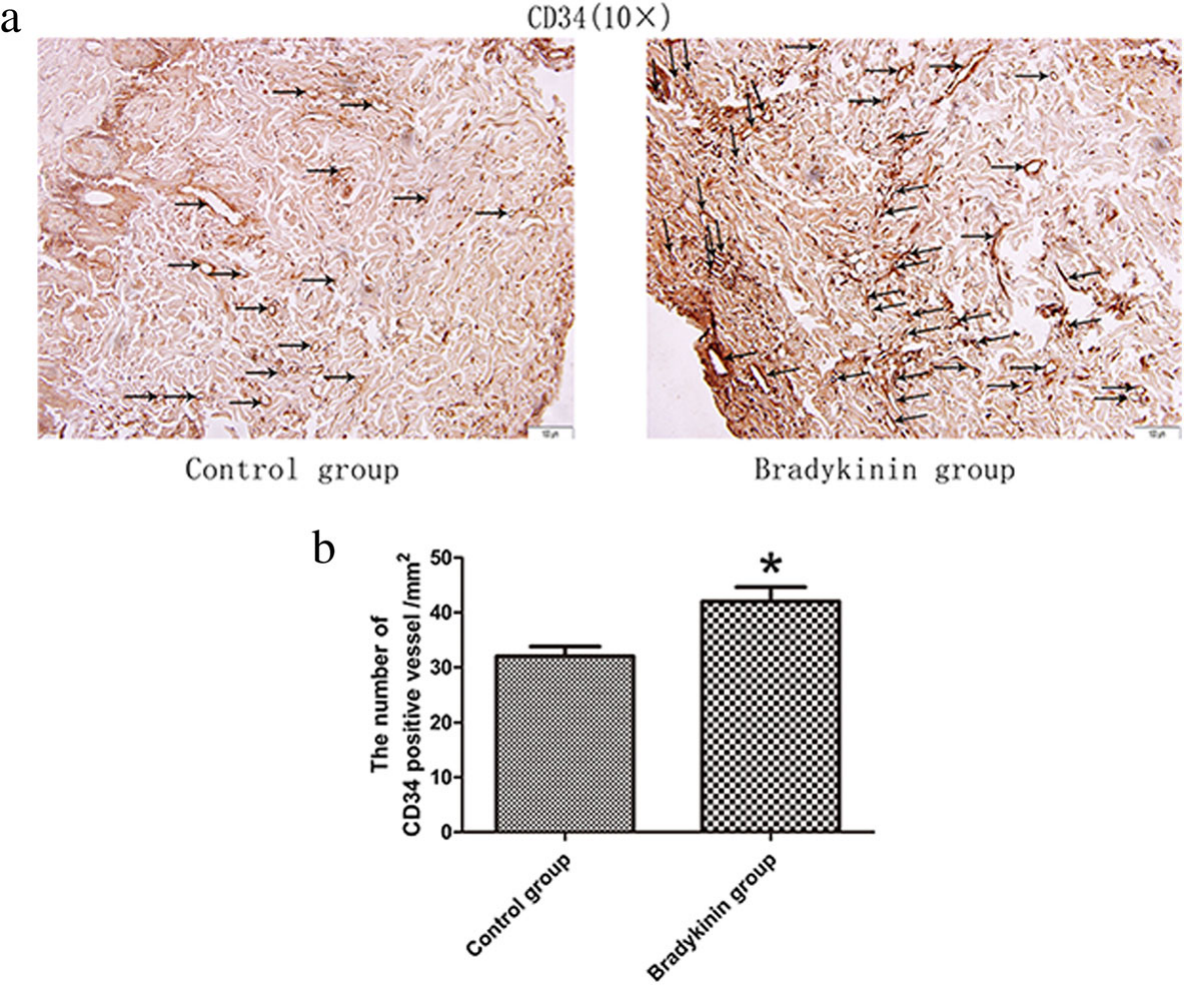
**a** The CD34-positive microvessels were represented by black arrows (original magnification × 100). **b** The number of CD34-positive vessels/mm^2^ was 42.13 ± 2.59/mm^2^ in the bradykinin group and 31.92 ± 1.40/mm^2^ in the control group. *n* = 5 per group. *P < 0.05

The flaps from rats treated with bradykinin showed more neovascularization and less necrosis than those from the control group (H&E staining; Fig. 2a). The mean MVD in the CVZ was higher in flaps from the bradykinin group than the control group (39.47 ± 1.35 vs. 30.38 ± 2.10, respectively; *P* < 0.05; Fig. 2b). Endothelial cells can be labeled by CD34. The number of CD34-positive vessels/mm^2^ can indicate the mean MVD. Immunohistochemistry staining revealed that the number of CD34-positive vessels was higher in the bradykinin group than the control group (42.13 ± 2.59/mm^2^ vs. 31.92 ± 1.40/mm^2^, respectively; *P* < 0.05; Fig. 3a, b).

### Western blot assay for VEGF

**Fig. 4:**
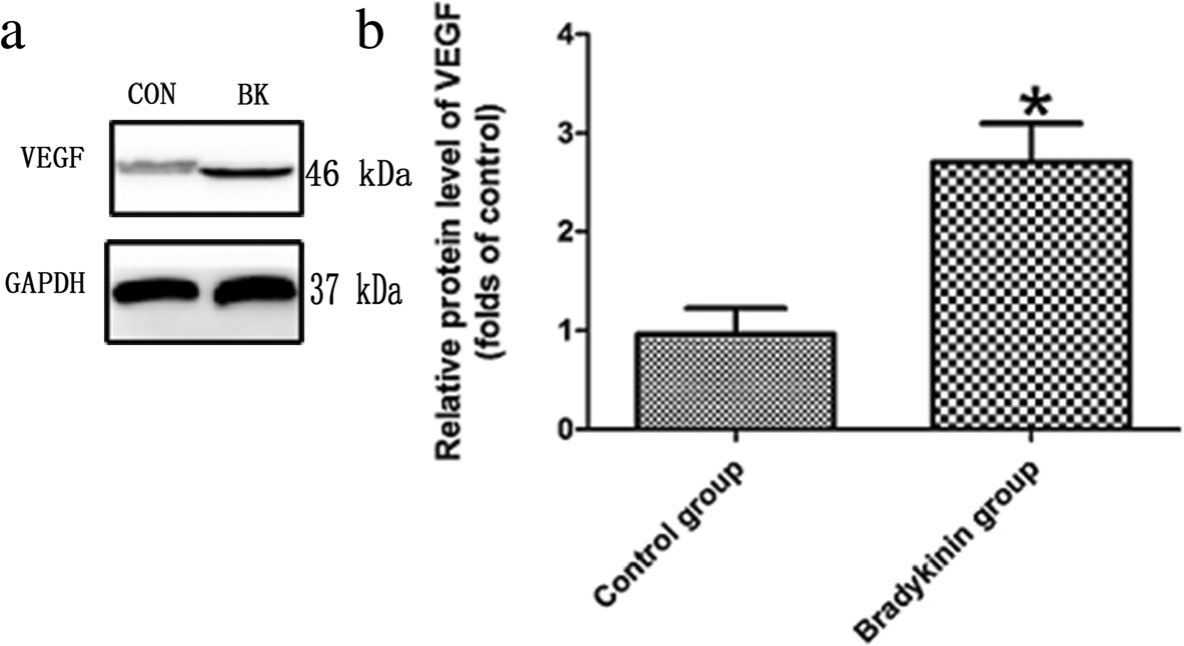
**a** Levels of VEGF protein in the choke vessel zone (CVZ) were calculated as the fold relative to the control. GAPDH served as the loading control. **b** The level of VEGF. *n* = 5 per group. *P < 0.05

The expression of VEGF in the CVZ of all perforator flaps was revealed by western blotting (Fig. 4a). VEGF expression was higher in the bradykinin group (*P* < 0.05; Fig. 4b).

### Western blot analyses of the apoptotic index

**Fig. 5:**
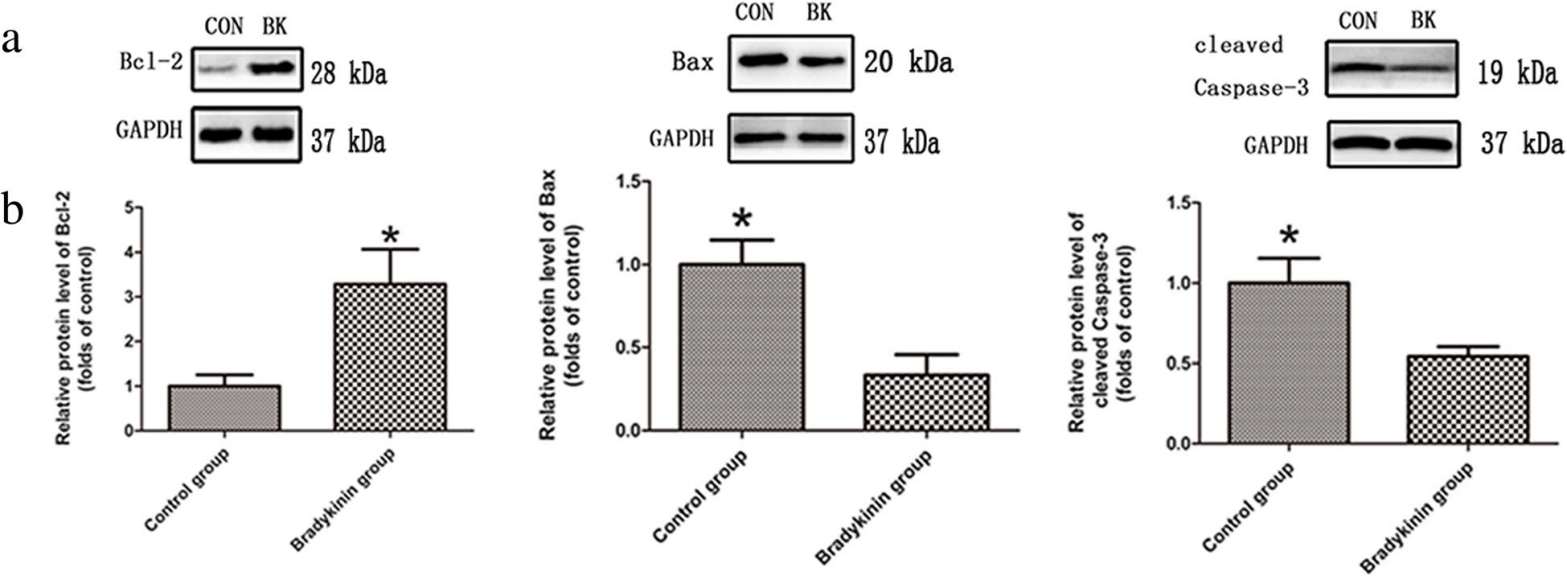
**a** Expression of apoptotic proteins (cleaved caspase-3, Bax, and BCL-2). **b** The relative protein levels of apoptotic proteins. Expression of apoptotic proteins were evaluated by optical density analyses, calculated as the fold relative to the control, and normalized to GAPDH. *n* = 5 per group. *P < 0.05

The expression of apoptotic proteins, including cleaved caspase-3, Bax, and Bcl-2, was investigated. Cleaved caspase-3 and Bax are two types of apoptotic proteins that participate in apoptosis, whereas Bcl-2 can resist apoptosis [20, 21]. The levels of cleaved caspase-3 and Bax were decreased, whereas that of BCL-2 was increased on POD 7 in the bradykinin group (*P* < 0.05; Fig. 5a, b).

### SOD and MDA content

**Fig. 6:**
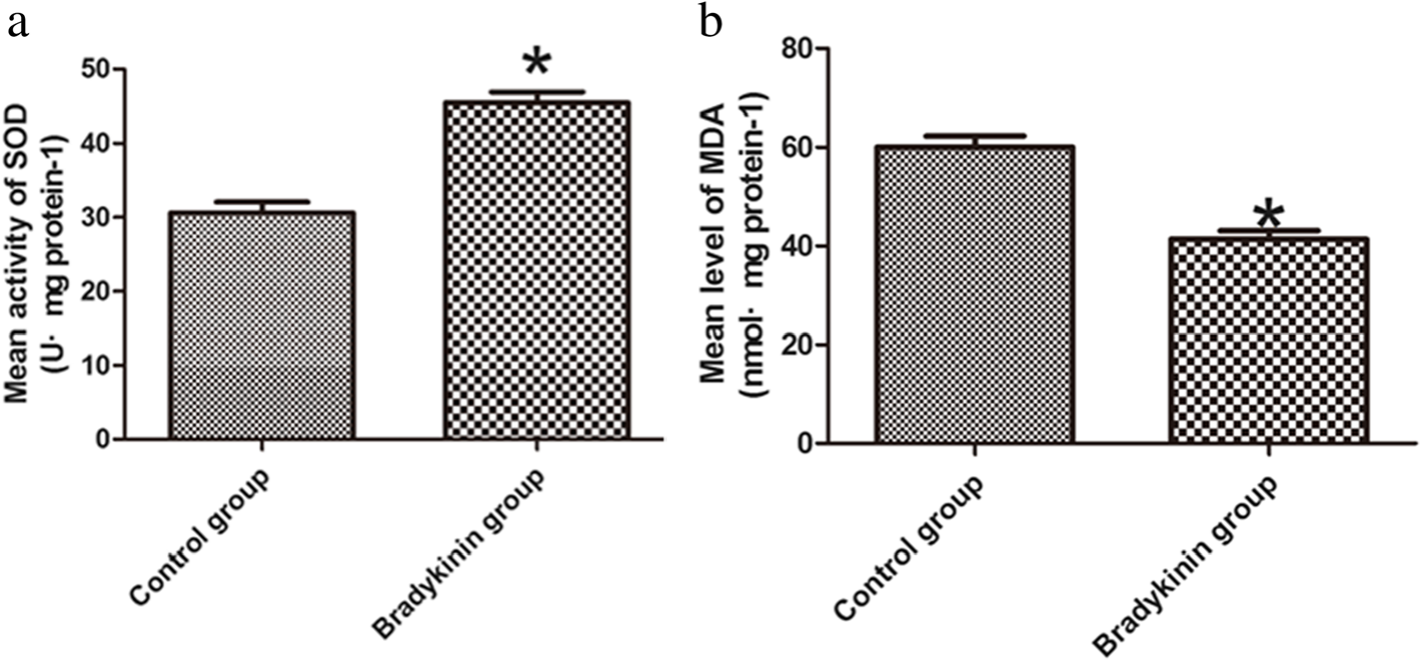
**a** SOD activity (U mg^−1^ protein^−1^). Bradykinin group, 45.46 ± 1.43; control group, 30.61 ± 1.47. **b** MDA content (nmol mg protein^−1^). Bradykinin group, 41.39 ± 1.67; control group, 60.05 ± 2.25. *n* = 5 per group. *P < 0.05

Compared to the control group, mean SOD activity was much higher in the bradykinin group (45.46 ± 1.43 U mg protein^−1^ vs. 30.61 ± 1.47 U mg protein^−1^, respectively; *P* < 0.05; Fig. 6a). The bradykinin group also had a much lower mean level of MDA (41.39 ± 1.67 nmol mg protein^−1^) (60.05 ± 2.25 nmol mg protein^−1^; *P* < 0.05; Fig. 6b).

### Western blot analyses of autophagy markers

**Fig. 7:**
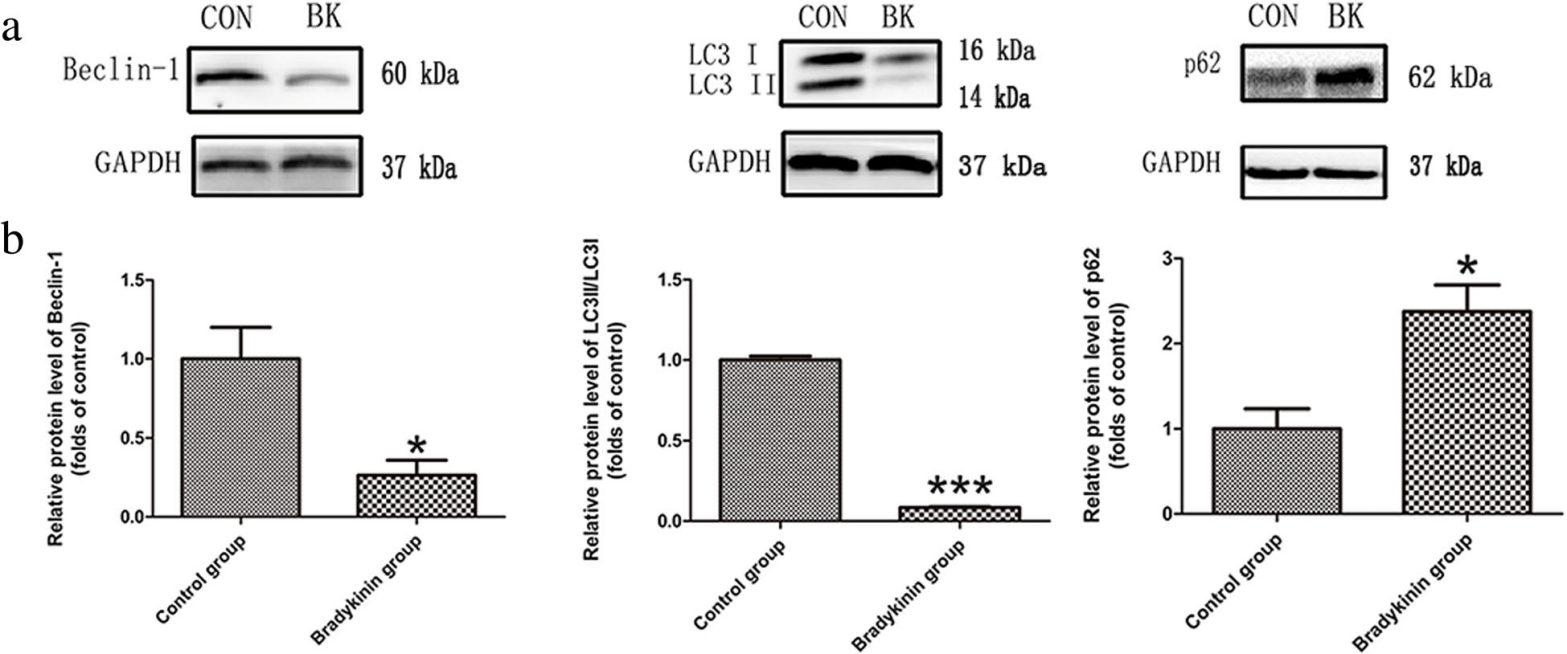
**a** Expression of p62, Beclin 1, and LC3. The intensity of band was calculated as the fold relative to the control and normalized to GAPDH. **b** Densitometric analyses of p62, the ratio of LC3 II to LC3 I, and Beclin 1. *n* = 5 per group. *P < 0.05, ***P < 0.001

Western blot was used to assess Beclin 1 and LC3II/LC3I expression in the CVZ of all perforator flaps. The ratio of LC3 II to LC3 I was downregulated as well as Beclin 1 in the bradykinin group. The control group had a higher LC3II/LC3I ratio and Beclin 1 expression than the bradykinin group (*P* < 0.05; Fig. 7a). p62 is a marker of autophagic flux. Expression of p62 was much higher in the bradykinin group than the control group (*P* < 0.05; Fig. 7b).

### NO content

**Fig. 8:**
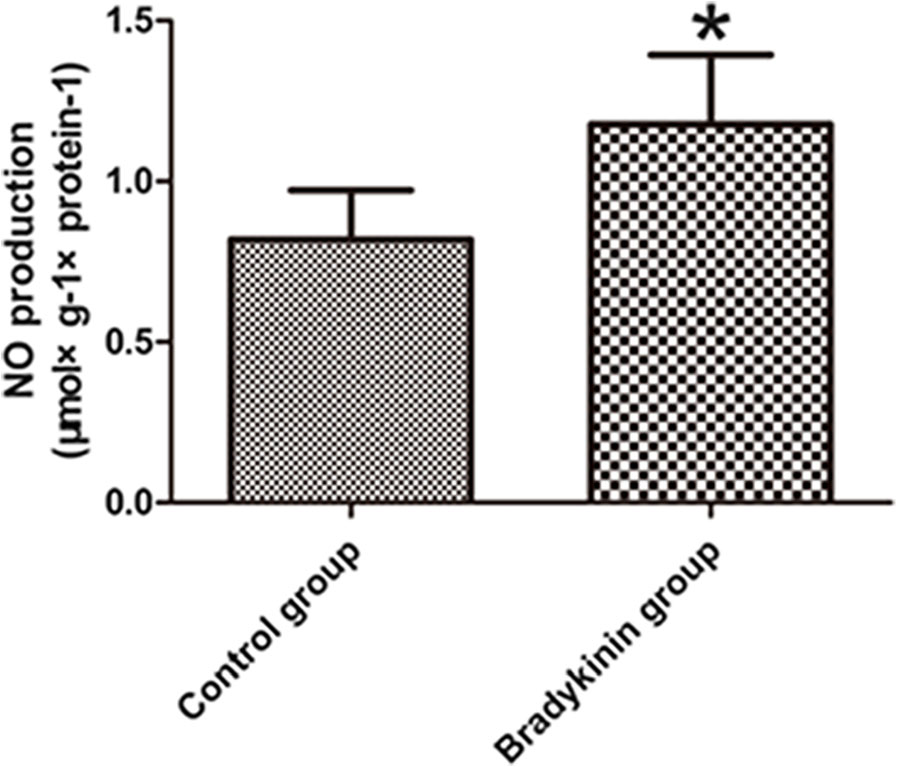
Nitric oxide (NO) production in the CVZ on POD 7. *n* = 5 per group. *P < 0.05

Compared to the control group, NO production was higher in the bradykinin group (1.18 ± 0.22 vs. 0.82 ± 0.15, respectively; *P* < 0.05; Fig. 8).

## Discussion

Bradykinin, a vasoactive peptide, is produced by the protease degradation of high molecular weight or low molecular weight kininogens [29]. Bradykinin plays important roles in both acute and chronic inflammatory processes. However, it also has anti-apoptotic [30] and antioxidative [31] properties and promotes vascularization [32]. Thus, we hypothesized that bradykinin would improve the viability of multiterritory perforator flaps by promoting vascularization, suppressing apoptosis, inhibiting autophagy, promoting the release of NO, and reducing oxidative stress.

Several authors have reported that treatment with bradykinin increases VEGF expression in tumors [33]. VEGF can promote angiogenesis [13]. In a kininogen-deficient rat tumor model (which cannot intrinsically generate bradykinin), expression of VEGF and the extent of angiogenesis were significantly less than in normal rats [34]. In skin flaps, VEGF which is particularly active in dermal vascular structures is secreted by fibroblasts and keratinocytes in the cutis [35]. VEGF can also promote vascularization in multiterritory perforator flaps [27]. In our study, the VEGF protein levels were upregulated after treatment with bradykinin. Western blot analyses also showed lower VEGF levels in the control group than the bradykinin group. In addition, MVD showed more neovascularization in the bradykinin group relative to the control group. So, we know that bradykinin promotes vascularization in multiterritory perforator flaps by improving VEGF expression.

A postoperative flap that suffers from I/R injury exhibits increased production of ROS, apoptosis, inflammation, and so forth [36, 37]. Several publications have described effective therapeutic strategies for inhibiting I/R injury [36–38]. Among them, the use of hyperbaric oxygen to inhibit I/R injury is accepted both clinically and experimentally [39, 40]. Flap survival is further improved by combining treatment with hyperbaric oxygen and vascular growth factor [41, 42]. As mentioned previously, the pathophysiological action of I/R injury determined the final outcome of the flap.

Apoptosis plays a vital role in flap survival. The I/R process can induce apoptosis which can lead to cell death [43]. Burns et al. reported that cellular apoptosis can lead to I/R injury [43]. With less apoptosis, flaps exhibit a better blood supply and larger survival area [44]. When apoptosis is exacerbated, attenuation of apoptosis ameliorates healing. Bradykinin had an anti-apoptotic effect under conditions of diabetes [45]. Our results also show that bradykinin inhibits apoptosis, as reflected by increased BCL-2 levels and decreased Bax levels [46, 47]. We also found that cleaved caspase-3 activity was reduced in the bradykinin group [48]. So, we reach the conclusion that bradykinin can suppress apoptosis in a multiterritory perforator flap.

I/R injury can produce ROS, a significant component of the complex oxidation process. ROS include free radicals, oxygen ions, and peroxides that initiate I/R damage [49–51]. ROS formed during I/R injury cause a lot of changes that damage microcirculation (e.g., swelling of endothelial cells, vasoconstriction) [37]. When skin flaps experience ischemia, the oxidase system will be upregulated significantly [52, 53]. This system is an important source of ROS production during ischemia [54, 55]. Inhibition of the xanthine oxidase system reduces the formation of ROS and increases the survival rate of flaps [53, 56]. MDA content and SOD activity are biomarkers of oxidative stress. In our research, we used MDA content and SOD activity to assess oxidative stress. Bradykinin has a cardioprotective effect in acute cardiac I/R injury [57]. In this research, the MDA content was much lower in the bradykinin group relative to the control group, whereas SOD activity was higher. Thus, bradykinin suppresses oxidative stress in multiterritory perforator flaps.

ROS can induce angiogenesis but an excessive amount of ROS inhibits angiogenesis [58–60]. It remains to be determined about the effects of ROS on angiogenesis. These effects are likely dependent on different characteristics of a disease. In the present study, bradykinin improved the survival of multiterritory perforator flaps by suppressing oxidative stress and accelerating vascularization. However, the relationship between oxidative stress and vascularization in multiterritory perforator flaps after treatment with bradykinin remains to be confirmed.

Scherz–Shouval and Elazar previously reported that ROS induces autophagy [61]. By contrast, Vande et al. reported that excessive autophagy increased the level of ROS production [62]. Excessive autophagic activity has a detrimental effect. It can consume functional components under conditions of excessive autophagy and promote cell death [20, 63]. For instance, many experts have reported that excessive autophagy enhances apoptosis, ROS production, and tissue injury [62–64]. The effects of autophagy vary by disease or different periods of a disease [65]. Wang et al. reported that inhibiting autophagy enhanced perforator flap survival [8]. In the current study, bradykinin also regulated autophagy levels. Western blot analyses revealed that expression of LC3II/LC3I and Beclin 1 decreased, which indicates that autophagy was inhibited in the bradykinin group. p62 is an autophagic flux marker. p62 was increased in the group treated with bradykinin, which indicates that autophagy was inhibited. Thus, bradykinin likely downregulates the level of autophagy in multiterritory perforator flaps.

Several authors have reported that bradykinin can activate the PI3K/Akt signaling pathway [18, 24, 66]. This signaling pathway promotes the release of NO [22, 23]. Low levels of NO inhibit PTEN activity, thereby stimulating the Akt signaling pathway, which suppresses neuronal apoptosis [67]. Inactivation of PTEN results in increased mTORC1 activity, leading to the inhibition of autophagy [19]. NO, the product of NOS, promotes cell proliferation and angiogenesis [25], which is also beneficial for flap survival. In our study, NO levels decreased in the control group compared to the bradykinin group. Thus, NO inhibits apoptosis, autophagy, and tissue injuries. From the results, we conclude that bradykinin suppresses apoptosis and autophagy by promoting the release of NO in perforator flaps. The function of NO in angiogenesis is also important for flap survival.

## Conclusion

Bradykinin increased angiogenesis, suppressed apoptosis, inhibited autophagy, and suppressed oxidative stress, leading to higher perforator flap survival rate. In addition, NO production was promoted in perforator flaps treated with bradykinin, which may suppress apoptosis and inhibit autophagy.

## Abbreviations

BK: Bradykinin
CON: Control
CST: Cell Signaling Technology
CVZ: Choke vessel zone
DCI: Deep circumflex iliac
DCIA: Deep circumflex iliac artery
H&E: Hematoxylin and eosin
I/R: Ischemia–reperfusion
IC: Intercostal artery
IHC: Immunohistochemistry
MDA: Malondialdehyde
MVD: Microvascular density
NO: Nitric oxide
NOS: Nitric oxide synthase
POD: Postoperative day
PU: Perfusion units
SEMs: Standard errors of the mean
SOD: Superoxide dismutase
TD: Thoracodorsal artery
VEGF: Vascular endothelial growth factor
